# Sperm Motility of Mice under Simulated Microgravity and Hypergravity

**DOI:** 10.3390/ijms21145054

**Published:** 2020-07-17

**Authors:** Irina V. Ogneva, Maria A. Usik, Nikolay S. Biryukov, Yuliya S. Zhdankina

**Affiliations:** 1 Cell Biophysics Laboratory, State Scientific Center of the Russian Federation, Institute of Biomedical Problems of the Russian Academy of Sciences, 76a, Khoroshevskoyoe Shosse, Moscow 123007, Russia; usik.maria@mail.ru (M.A.U.); lboy90@yandex.ru (N.S.B.); juliaszd@yandex.ru (Y.S.Z.); 2 Department of Medical and Biological Physics, I. M. Sechenov First Moscow State Medical University, 8-2 Trubetskaya Street, Moscow 119991, Russia

**Keywords:** sperm motility, cell respiration, cytoskeleton, simulated microgravity, hypergravity

## Abstract

For deep space exploration, reproductive health must be maintained to preserve the species. However, the mechanisms underlying the effect of changes in gravity on male germ cells remain poorly understood. The aim of this study was to determine the effect of simulated micro- and hypergravity on mouse sperm motility and the mechanisms of this change. For 1, 3 and 6 h, mouse sperm samples isolated from the caudal epididymis were subjected to simulated microgravity using a random position machine and 2g hypergravity using a centrifuge. The experimental samples were compared with static and dynamic controls. The sperm motility and the percentage of motile sperm were determined using microscopy and video analysis, cell respiration was determined by polarography, the protein content was assessed by Western blotting and the mRNA levels were determined using qRT-PCR. The results indicated that hypergravity conditions led to more significant changes than simulated microgravity conditions: after 1 h, the speed of sperm movement decreased, and after 3 h, the number of motile cells began to decrease. Under the microgravity model, the speed of movement did not change, but the motile spermatozoa decreased after 6 h of exposure. These changes are likely associated with a change in the structure of the microtubule cytoskeleton, and changes in the energy supply are an adaptive reaction to changes in sperm motility.

## 1. Introduction

During deep space exploration, obtaining healthy offspring of higher animals and maintaining the reproductive health of individuals of different sexes are nontrivial tasks. During the space flight of the Cosmos-1129 biosatellite, effective mating of rats could be achieved, but pregnancy did not occur [1]. Abortion of preimplantation embryos in mice was also observed [2]. In an in vitro experiment, 49 two-cell mouse embryos on board the Columbia Space Shuttle (STS-80) were assessed: none of the embryos developed [3].

The reasons for these failures could be related both to the difficulties of conducting an experiment in space flight conditions and to the characteristics of the early embryogenesis of mammals and the maturation of their germ cells. For example, under simulated microgravity conditions by a random positioning machine, a decrease in the survival of mouse follicles was observed with aberrant development of granulosa cells and oocytes, as shown by a reduction in the content of related markers [4].

Male germ cells represent a specific target for the action of microgravity, as movement and capacitation of these cells are necessary for successful natural fertilization.

Interestingly, the spermatozoa of lower animals (sea urchin) were examined in space experiments as part of the space shuttle expeditions STS-81 (January 1997) and STS-84 (May 1997), and phosphorylation of the phosphothreonine-containing protein FP130 occurred three–four times faster under microgravity conditions than under 1g conditions, indicating more intense activation of sperm motility, since it is associated with a change in phosphorylation level [5].

Storage of mouse sperm at −90 °C on the ISS, where the background radiation is 100 times higher than that at ground level, for nine months led to an increase in DNA damage; however, fertilization and fertility were similar to those of the control group. Sequencing of the next generation showed only slight genomic differences between offspring derived from sperm stored in space and the control sperm, and all offspring grew to maturity and had normal fertility [6].

However, space flight conditions reduce the number of mature spermatozoa in the epididymides, and in model experiments, such as those with antiorthostatic suspension, a significant decrease occurred [1,7,8]. This decrease may be due to the combined action of multiple factors, namely a change in the structural and functional state of the progenitor cells and a change in blood flow in the male genitals. Moreover, it is unlikely that such disorders are due to changes in hormonal status. A study carried out after a six-week antiorthostatic suspension of male rats showed that the testes mass and spermatogenesis were reduced; spermatogenous cells were not observed, with the exception of round spermatids; and accordingly, no mature sperm was observed in the epididymis. Germ cells were also absent in the duct deferens, with the exception of several spermatogonia, which was associated with a change in Sertoli cells. However, the levels of testosterone, luteinizing hormone and follicle-stimulating hormone did not change [9].

After a 30-day antiorthostatic suspension of mice, we observed various changes in the spermogram of the mice: a decrease in the number of motile spermatozoa, a significant decrease in the proportion of viable sperm and a normal sperm morphology. According to the data on the sperm-specific protein levels, there was a shift towards immature forms, as well as various changes in the content of cytoskeletal proteins and the expression of the corresponding genes [10].

Thus, microgravity conditions lead to multiple structural and functional changes in native germ cells, especially male germ cells. However, the pathogenesis of the changes in response to a mechanical stimulus remains unclear. Therefore, in this study, we focused on the sperm motility of mice during their short-term cultivation in altered mechanical conditions: simulated microgravity and hypergravity (2g).

## 2. Results

We used the following notation for experimental groups: static control (CS), dynamic control (CD), simulated microgravity group (s-μg) and hypergravity group (2g) for each time point (1, 3 and 6 h) described in detail in the Section 4 (see below).

### 2.1. Sperm Motility

**Figure 1:**
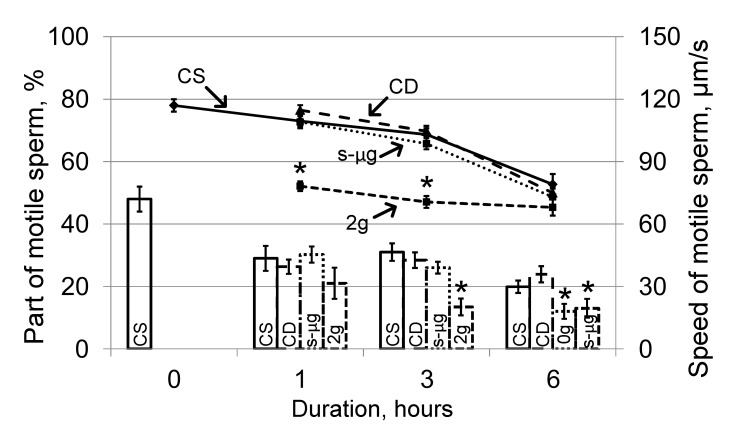
Portion of motile sperm and the speed after 1, 3 and 6 h of exposure under simulated microgravity and hypergravity (2g). The histogram shows the proportion of motile sperm in each sample (left axis), graph—change in the speed of movement (right axis). CS—static control, CD—dynamic control, s-μg—simulated microgravity conditions, 2g—hypergravity conditions. * *p* < 0.05 compared with the static control group at the same time point. The data are presented as the mean ± standard error of the mean (M ± SE).

Cultivation of sperm in the medium led to a decrease in the number of motile spermatozoa and a decrease in the speed of movement in the static control group relative to the zero control group. The proportion of motile spermatozoa was 29 ± 4% in the CS1 group, 31.0 ± 2.8% in the CS3 group, 19.9 ± 2.0% in the CS6 group and 48 ± 4% in the CS0 group (histogram, Figure 1). The speed of movement after 1 and 3 h did not significantly differ from that at the zero point (CS1, 109.5 ± 2.4 μm/s; CS3, 103 ± 3 μm/s; compared to 117 ± 3 μm/s in CS0). After 6 h, the speed significantly decreased by 32% (*p* < 0.05) and was 79 ± 5 μm/s in the CS6 group (Figure 1, graph). The motility parameters in the dynamic control groups did not differ from those in the corresponding static control groups; therefore, the motility parameters in the s-μg and 2g groups were compared with those in the corresponding static control groups.

Exposure under the simulated microgravity conditions led to significant changes in the portion of motile spermatozoa after 6 h: it decreased almost two times compared with that of the corresponding control (histogram, Figure 1), although the speed did not change (graph, Figure 1).

Cultivation in hypergravity conditions led to a decrease in the speed of sperm movement by 29% (*p* < 0.05) relative to that of the corresponding control group at 1 h (78.2 ± 2.3 μm/s vs. 109.5 ± 2.4 μm/s), and the absolute values of speed in the group with hypergravity exposure did not change. Against the background of a decrease in speed in the control groups during cultivation, after 6 h, the speed of movement in the 2g6 and CS6 groups did not differ from each other (graph, Figure 1). The proportion of motile spermatozoa under hypergravity decreased relative to that of the control after 3 h of exposure and remained at the same level after 6 h (histogram, Figure 1).

### 2.2. Cell Respiration

At the end of the measurement, each of the samples was subjected to a mitochondrial membrane test by adding 10 mM cytochrome *c*. All analyzed samples after permeabilization had an intact membrane. At all time points, the rate of cell respiration between the static and dynamic controls was not significantly different.

**Figure 2:**
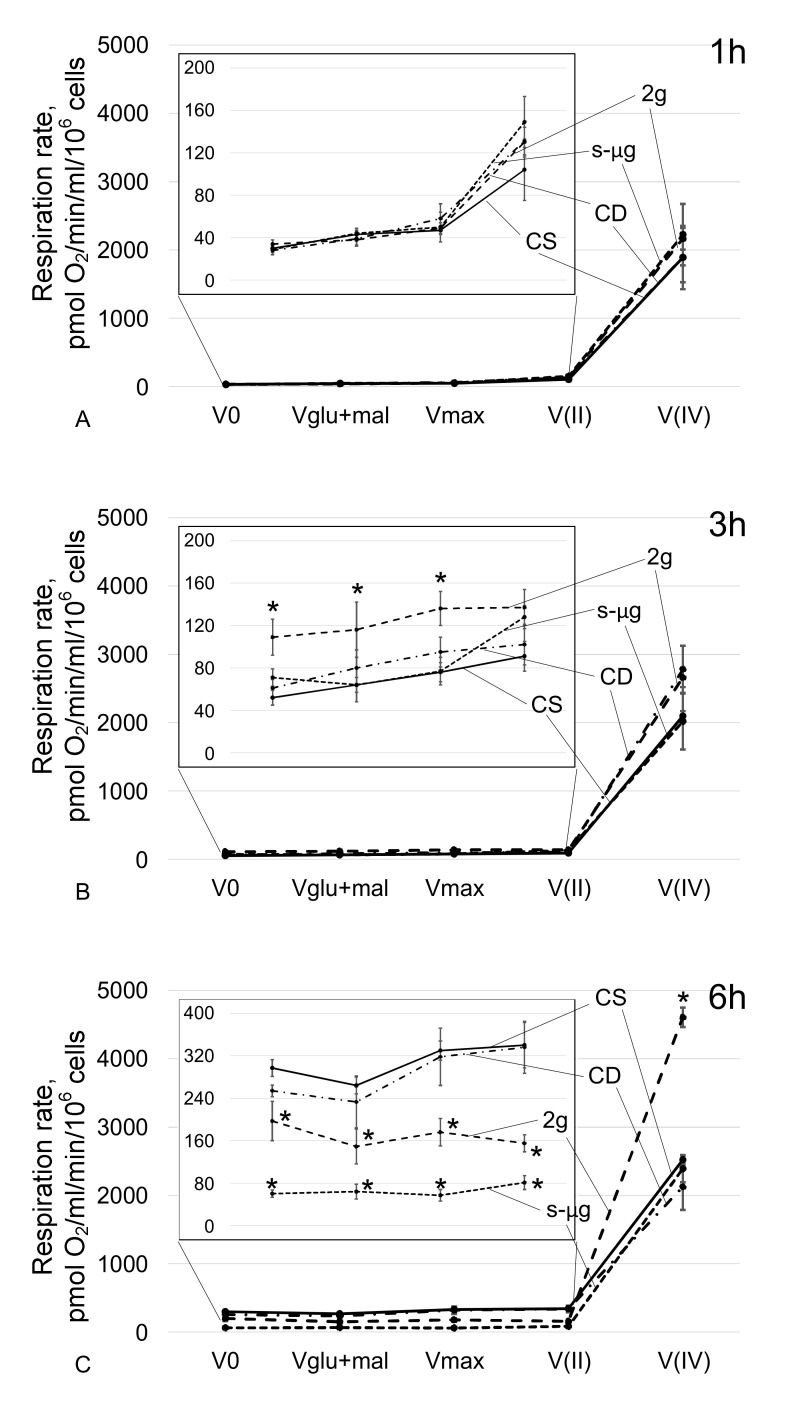
Absorption rate of oxygen by sperm after cultivation under simulated microgravity and hypergravity conditions. (**A**)—cultivation for 1 h; (**B**)—cultivation for 3 h; (**C**)—cultivation for 6 h. CS—static control, CD—dynamic control, s-μg—simulated microgravity conditions, 2*g*—hypergravity conditions. V0—respiration rate of permeabilized cells; Vglu+mal—respiration rate after addition of 10 mM glutamate + 5 mM malate; Vmax—maximum respiration rate after addition of 2 mM ADP; V(II)—respiration rate after addition of 0.5 mM rotenone (complex I inhibitor) and then 10 mM succinate (substrate of complex II); V(IV)—respiration rate after addition of 5 mM antimycin (complex III inhibitor) and then 0.5 mM TMPD + 2 mM ascorbate (artificial substrates of complex IV). The change in respiration rate from V0 to V(II) in increased resolution is presented on the graphs in the frame. * *p* < 0.05 compared with the corresponding static control group. The data are presented as the mean ± standard error of the mean (M ± SE).

**Figure 3:**
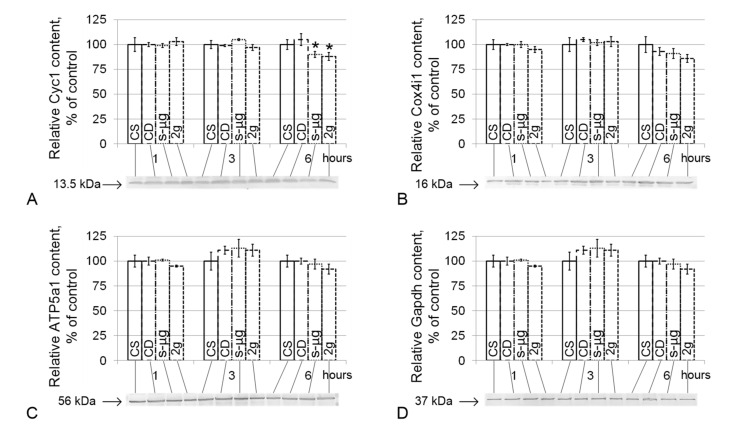
Relative levels of proteins involved in cell respiration. (**A**)—Cyc1, cytochrome *c*-1 (13.5 kDa, protein of the respiratory chain, located between complexes III and IV); (**B**)—Cox4i1, cytochrome *c* oxidase (16 kDa, protein of complex IV of the respiratory chain); (**C**)—ATP5a1, ATP synthase F1 (56 kDa, subunit of ATP synthase); (**D**)—Gapdh, glyceraldehyde-3-phosphate dehydrogenase (37 kDa, catalyzes one step of the glycolytic breakdown of glucose). * *p* < 0.05 compared with the static control mean. Typical Western blots for each protein were performed and are shown in the histogram. The data are presented as the mean ± standard error of the mean (M ± SE).

When spermatozoa were cultured under simulated microgravity conditions for 1 h (Figure 2A) and 3 h (Figure 2B), changes in the rate of oxygen uptake were not observed, including during substrate-inhibitor analysis. After 6 h, V0, Vglu + mal, Vmax and V(II) significantly (*p* < 0.05) decreased by 80%, 76%, 83% and 76%, respectively, compared with those of the relatively static control (Figure 2C). However, V(IV) in the s-μg6 group did not differ from that in the CS6 group.

Under hypergravity conditions, after 1 h, the rate of oxygen uptake did not change (Figure 2A). After 3 h, V0, Vglu+mal and Vmax increased relative to the control level by 110%, 81% and 78% (*p* < 0.05), respectively (Figure 2B). In this case, V(II) and V(IV) did not change. In the 2g6 group, V0, Vglu + mal, Vmax and V(II) decreased by 34%, 44%, 47% and 54% (*p* < 0.05), but V(IV) increased by 83% (*p* < 0.05) compared with similar parameters in the CS6 group (Figure 2C).

### 2.3. Protein Relative Content

The relative content of all the studied proteins did not significantly differ between the corresponding groups of static and dynamic controls.

The relative content of cytochrome *c*-1 (Cyc1) slightly, although significantly, decreased in the simulated micro- and hypergravity conditions after 6 h by 10% in the s-μg6 group (*p* < 0.05) and by 12% in the 2g6 group (*p* < 0.05) compared with the CS6 group (Figure 3A). The contents of cytochrome *c* oxidase (Cox4i1), ATP synthase F1 (ATP5a1) and glyceraldehyde-3-phosphate dehydrogenase (Gapdh) did not differ from the corresponding control levels either under simulated microgravity or hypergravity conditions (Figure 3B–D).

**Figure 4:**
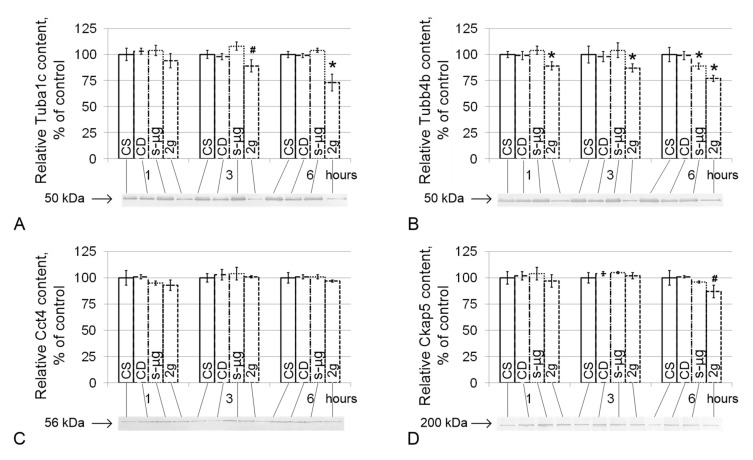
The relative protein levels of components of the microtubule cytoskeleton that participate in sperm tail movement. (**A**)—Tuba1c, alpha-tubulin (50 kDa, component of the tubulin heterodimer); (**B**)—Tubb4b, beta-tubulin (50 kDa, component of the tubulin heterodimer); (**C**)—Cct4, chaperonin containing Tcp1 subunit 4-delta (56 kDa, participates in the assembly of tubulin heterodimers); (**D**)—Ckap5, cytoskeleton-associated protein 5 (200 kDa, binds microtubules to each other and to the membrane). * *p* < 0.05, ^#^ *p* < 0.1 compared with the static control group. Typical Western blots for each protein are shown in the histogram. The data are presented as the mean ± standard error of the mean (M ± SE).

The relative content of alpha-tubulin (Tuba1c) did not differ among the CS, CD and s-μg groups (Figure 4A), and in group 2g, it decreased by 11% after 3 h (*p* < 0.1) and by 27% after 6 h (*p* < 0.05). The content of the second microtubule component, beta-tubulin (Tubb4b), in the 0g group decreased by 13% (*p* < 0.05) after 6 h (Figure 4B). However, in the 2g group, a significant decrease in the relative content of Tubb4b was noted after 1 h (by 11%, *p* < 0.05), after 3 h (by 13%, *p* < 0.05) and after 6 h (by 23%, *p* < 0.05).

The relative content of chaperonin containing Tcp1 subunit 4-delta (Cct4) did not change in the CS, CD, s-μg and 2g groups (Figure 4C); the content of cytoskeleton-associated protein 5 (Ckap5) decreased by 13% (*p* < 0.1) only in the 2g6 group (Figure 4D).

### 2.4. Relative mRNA Content

The relative content of all mRNAs did not significantly differ between the corresponding groups of static and dynamic controls at each time point.

**Figure 5:**
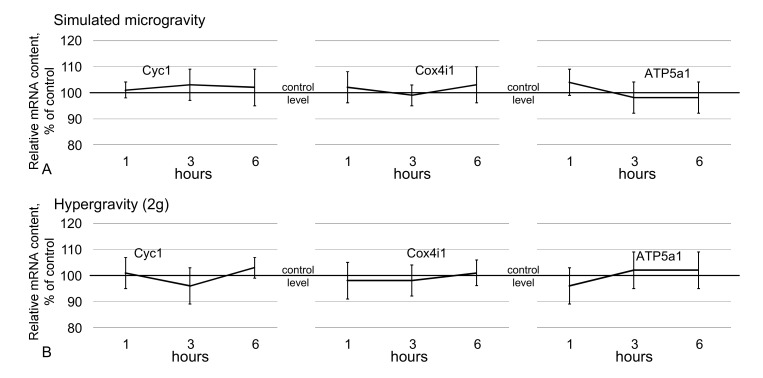
Relative mRNA content of genes encoding the analyzed metabolic proteins. (**A**)—genes encoding metabolic proteins under simulated microgravity; (**B**)—genes encoding metabolic proteins under hypergravity (2g). The data are presented as the mean ± standard error of the mean (M ± SE).

In addition to the protein content, the relative mRNA content of genes encoding proteins involved in cell respiration (cytochrome *c*-1, cytochrome *c* oxidase, ATP synthase F1) did not differ from the control level up to 6 h of exposure in simulated microgravity (Figure 5A) and hypergravity conditions (Figure 5B).

**Figure 6:**
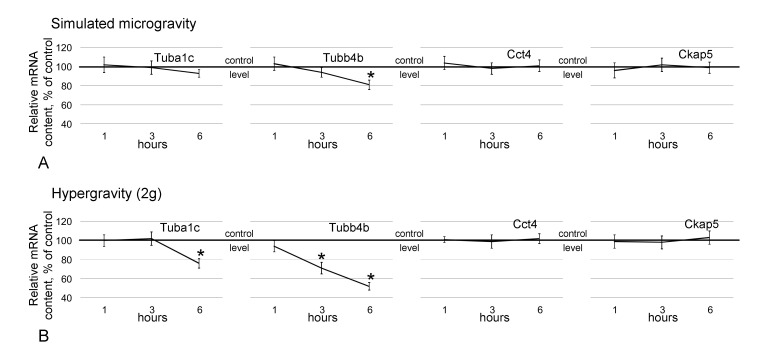
Relative mRNA content of genes encoding the analyzed cytoskeletal proteins. (**A**)—genes encoding cytoskeletal proteins under simulated microgravity; (**B**)—genes encoding cytoskeletal proteins under hypergravity (2g). * *p* < 0.05 compared with the static control group. The data are presented as the mean ± standard error of the mean (M ± SE).

The relative Tuba1c mRNA level did not differ between the CS, CD and s-μg groups, but in the 2g group, it decreased by 24% (*p* < 0.05) after 6 h compared with the corresponding control level (Figure 6B). The content of Tubb4b under simulated microgravity conditions decreased after 6 h by 19% (*p* < 0.05) compared with the control level (Figure 6A); under hypergravity conditions, after 3 h, the level was decreased by 29% (*p* < 0.05), and after 6 h, it was decreased by 48% (*p* < 0.05) relative to the control level (Figure 6B). The relative mRNA levels of Cct4 and Ckap5 did not change among the CS, CD, s-μg and 2g groups (Figure 6) up to 6 h of exposure.

## 3. Discussion

Analysis of the regulation of sperm motility in mammals under changing external mechanical conditions is important to prevent possible future reproductive problems after or during long-term space missions. The mechanism of triggering various changes at the cellular level in zero gravity is still obscure. Therefore, in this work, using an increase and decrease in external mechanical stress, we analyzed the mechanisms underlying the changes in the motility of mouse sperm.

It should be noted that simulated microgravity is not real microgravity. The principle of the random positioning machine is that there is a change in the orientation of the cells relative to gravity in such a way that the superposition of the vectors per minute is equal to zero on average. The fundamental difference with real weightlessness lies in such an alternating impact. However, it should be noted that even in a real space flight, at least in low Earth orbit, it is almost impossible to achieve zero gravity due to a number of technogenic factors, for example, turning on the engines on the ISS to correct the orbit. Moreover, short-term experiments aimed at identifying the mechanisms of cell signaling in real space flight are extremely difficult. Therefore, by far the most widely used method is simulated microgravity.

Changes in sperm motility were more pronounced under hypergravity than under microgravity, which is consistent with data obtained on human sperm in parabolic flights [11]. A decrease in speed under 2g conditions was observed after 1 h, and after 3 h, the number of motile cells begins to decrease. Moreover, under the conditions of simulated microgravity, the speed of movement relative to that of the corresponding controls did not change, but the number of motile spermatozoa significantly decreased after 6 h.

The motor activity of sperm is determined by the state of the microtubule cytoskeleton, which provides motility, and its energy supply. Previous studies showed that a decrease in the mechanical load on skeletal muscle cells leads to a decrease in the efficiency of cell respiration [12,13,14,15,16] and an increase in the volume mechanical load, particularly on cardiomyocytes, to an increase in the rate of energy production [14]. Therefore, we hypothesized that the observed changes in motility may be associated with changes in cell respiration.

Surprisingly, we did not find changes in the rate of cellular respiration after 1 h of hypergravity conditions, and after 3 h, the respiration rate in the 2g group was significantly higher than that in the control. Inhibitor analysis indicated that this increase was due to the increased efficiency of complex I of the respiratory chain since when it was inhibited and substrate II of the complex was then added, the rates did not differ from that of the control. It should be noted that an increase in the activity of complex I of the respiratory chain could lead to the accumulation of ROS. As a result of electron leakage of the respiratory chain, a significant amount of ROS is formed as a by-product of oxidative phosphorylation. The main contribution is made by the I and III complexes of the respiratory chain, during which electron leakage leads to the reduction of oxygen to superoxide-ion [17]. Under normal conditions, superoxide is rapidly transformed into hydrogen peroxide due to the work of superoxide dismutase, and then it can be reduced to water by catalase or glutathione peroxidase [18]. It is well known that when preparing sperm for in vitro fertilization protocols, semen is separated by centrifugation. It was shown that a significant accumulation of ROS occurs in this case, which correlates with time and applied acceleration [19,20,21,22,23]. However, it should be noted, that the minimum value used in these protocols is 100 times higher than 2g. Nevertheless, a similar, albeit not so significant, effect cannot be ruled out. However, even this effect does not explain the increase in the rate of cellular respiration after 3 h against the background of a decrease in mobility, which was observed after 1 h.

A decrease in motility leads to a decrease in ATP consumption and, logically, should lead to a decrease in respiratory rate. However, the energy supply of sperm is mainly due to glycolysis [24]. At the early stages of adaptation to hypergravity conditions, transient ATP accumulation may occur due to a decrease in its utilization, and then, the Pasteur effect occurs: suppression of phosphofructokinase activity under the accumulation of ATP and a switch from glycolysis to oxidative phosphorylation; as a result, the rate of oxygen consumption is increased. Further, up to 6 h, the expected decrease in the rate of cell respiration occurred, both under conditions of hypergravity and simulated microgravity. Moreover, a decrease in the content of one of the key proteins of the respiratory chain, cytochrome *c*, was observed in the s-μg and 2g groups: this decrease is most likely due to its proteolysis, since the mRNA level did not change. Thus, given the results of cell respiration analysis, it is likely that the observed changes are an adaptation of the sperm energy supply system to a new regime of motor activity but not its cause. However, in analyses of cardiomyocytes, the intensity of cell respiration was shown to depend on the state of the cytoskeleton [25], as well as cell motility. Therefore, we further analyzed the content of proteins related to the microtubule cytoskeleton.

After 1 h of exposure under hypergravity conditions, the relative content of beta-tubulin, which is one of the subunits of the tubulin heterodimer of microtubules, decreased, and after 3 h, the content of the second subunit, alpha-tubulin, began to decrease. Notably, the decrease in the content of cytoskeletal proteins, as well as cytochrome *c*, is due to proteolysis since the content of the corresponding mRNA decreased later than the protein content. In the case of the accumulation of tubulin subunits, in contrast to the assembly of microtubules, their degradation may begin and subsequently affect the translation process, as well as the content of the corresponding mRNA [26,27]. However, the content of chaperonin containing Tcp1, subunit 4 (delta) Cct4, which ensures the assembly of newly synthesized tubulin alpha and beta subunits into heterodimers in the presence of magnesium ions and ATP [28], did not change throughout the entire exposure period. However, after 6 h, the relative content of the cytoskeleton-associated protein 5 Ckap5, which binds microtubules to each other and binds to the plus end, preventing their destruction [29], decreased.

The beta-tubulin content also decreased under simulated microgravity conditions but only after 6 h, when a decrease in the number of motile spermatozoa occurs. Microtubule networking is a gravitationally dependent process [30,31,32,33,34]. However, the issue of microtubule assembly under these conditions is still unclear. According to some reports, self-assembly of tubulin into microtubules occurs regardless of gravity [35,36]. In contrast, another report showed that after 10 days of cultivation of mesenchymal stem cells under simulated microgravity conditions, the polymerization activity of tubulin was not changed, but after three days, the authors observed decomposition of microtubules [37]. Nevertheless, it can be assumed that a decrease in the content of at least one of the components of the tubulin heterodimer forming the flagella axoneme will naturally lead to a change in sperm motility.

The results obtained indicate that even a very short exposure under altered mechanical conditions leads to changes in sperm, which, with longer exposure, will probably become more pronounced. On the one hand, this will make it possible to visualize, for example, the destruction of the cytoskeleton structure by staining in order to localize the changes. On the other hand, the number of factors acting inside the cell will increase. For example, oxidative stress and ROS accumulation can also lead to structural changes. In general, probable oxidative stress and destruction of the cytoskeleton can affect the fertilizing ability, which makes these changes critical for fertilization and early development, and during a long space flight—for maintaining the species. In addition, the influence of other factors of space exploration, especially beyond the Earth’s magnetosphere, such as radiation and hypomagnetic conditions, can also lead to negative consequences for reproductive function and requires further study.

## 4. Materials and Methods

### 4.1. Experimental Design

For sperm collection, we used both caudal epididymides from each male mouse and received sperm as described previously [10]. Briefly, immediately after extraction, the epididymis was placed in 1.2 mL α-MEM medium with 10% bovine serum heated to 37 °C. Then, it was ground and incubated on a shaker (Thermo Shaker PST-60HL-4, Biosan, South Korea) for 15 min at 37 °C. The resulting suspended material was filtered through nylon filters with a mesh diameter of 70 μm during 2 min.

All procedures conducted with animals were approved by the Commission on Biomedical Ethics of the Institute of Biomedical Problems (IBMP), the State Scientific Center of the Russian Federation and the Federal State Budgetary Institution of Science (Minutes No. 521 dated 25 September 2019).

**Figure 7:**
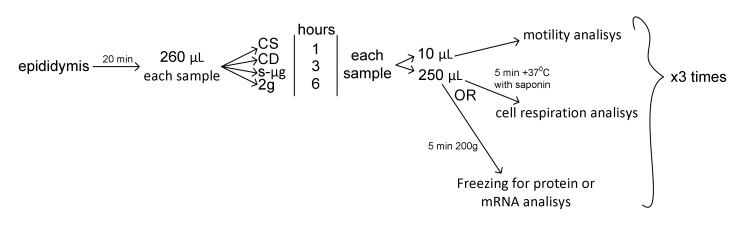
Experimental design and timeline of probe preparation.

After collection, cells from each epididymis were randomly divided into four groups for each time point and for each analysis (Figure 7). Next, there were designations: CS—static control, CD—dynamic control, s-μg—simulated microgravity group, 2g—hypergravity group. The number after the group designation means the time point.

Microgravity effects were modeled by using a random positioning machine [38], and hypergravity was simulated by using a centrifuge. We had static and dynamic controls (on a shaker with a similar velocity to the random position machine, but only in one direction) for each experimental group that was exposed in the same physical conditions.

After exposure, aliquots of the sperm from all groups were used for motility analysis. In the first round of the study, we measured cell respiration; in the second, protein content; and in the third, mRNA content. For protein and mRNA extraction, samples (after taking aliquots for motility analysis) were centrifuged during 5 min at 200× *g*, the supernatant was wasted and the precipitate was frozen in liquid nitrogen (Figure 7). For each time point and group, we had at least three biological replicas for each analysis.

### 4.2. Estimation of Sperm Motility

After exposure, 10 μL of the sperm suspension was applied to a Makler chamber (Sefi Medical Instruments, Ltd., Haifa, Israel) and observed under a phase-contrast microscope (Eclipse E200 MV, Nikon, Tokyo, Japan) with a magnification of 200× *g*. For sperm motility analysis, we made video recordings by a Basler puA1600-60uc color camera with an e2V EV76C570 CMOS sensor at 60 frames per second with a 2-megapixel resolution (Basler AG, Ahrensburg, Germany). For sperm motility analysis, we used computer software for semen analysis (MMCSperm Software, Saint-Petersburg, Russia) with settings for rodents. We estimated the proportion of motile sperm and their speed. To avoid any artifacts, we checked automatic data by manual counting of speed using the open software ImageJ with the tracking plugin in the Fiji software. To calculate the speed of movement (μm/s), we monitored the head of the mouse sperm, and the distance it traveled per second was measured.

### 4.3. Estimation of Cell Respiration by Polarography

For this method, for each experimental group, spermatozoa were isolated from the epididymis in α-MEM medium with 10% FBS as described previously [10], and the number of cells in the Makler chamber was estimated. Then, the permeabilization agent saponin was added at a concentration of 10 μg/mL, incubated for 5 min at 37 °C [39] and transferred to a polarographic chamber. Changes in the oxygen concentration were measured using an Oxygraph+ system (Hansatech Instruments Ltd., Norfolk, UK) at 22 °C.

Substrate-inhibitor analysis was performed according to the protocol described by Kuznetsov et al. [39]. After the sample was transferred to the polarographic chamber, V0, the rate of oxygen uptake by permeabilized cells, was recorded. Then, 10 mM glutamate and 5 mM malate, substrates of the first complex of the respiratory chain, were added, and the respiration rate was recorded as Vglu+mal. Next, 2 mM ADP was added, and the maximum respiration rate was recorded as Vmax. Then, inhibitors and substrates of the following respiratory chain complexes were alternately added to analyze their functional activity: 0.5 mM rotenone (complex I inhibitor), 10 mM succinate (complex II substrate; then, the oxygen absorption rate V(II) was recorded), 5 mM antimycin A (complex inhibitor III), 0.5 mM TMPD + 2 mM ascorbate (artificial substrates of complex IV; then, the oxygen absorption rate V(IV) was recorded). After substrate-inhibitor analysis, analysis for the intactness of the outer mitochondrial membrane was carried out for each sample by adding 10 mM cytochrome *c*: if the membrane was intact, the respiratory rate did not change or increased by a maximum of 15%. The cell respiration rate is expressed as pmol O_2_ per mL per min per cell count. We tested at least three biological replicas for each experimental time point.

### 4.4. Evaluation of Protein Content by Western Blotting

Frozen sperms were used for protein extraction. Cells were homogenized in Laemmli buffer containing a protease inhibitor cocktail (Calbiochem, San Diego, CA, USA). Denaturing electrophoresis on polyacrylamide gels was performed using the Laemmli method (Bio-Rad Laboratories, Hercules, CA, USA). Based on the measured concentration, an equal amount of protein was placed in each well, separated by electrophoresis and transferred to a nitrocellulose membrane [40]. The specific primary antibodies were used at the dilutions recommended by the manufacturers to determine the levels of proteins (Table 1).

Biotinylated goat antibodies were used as the secondary antibodies to detect mouse IgG (Sigma, Darmstadt, Germany, #B9904) at a dilution of 1:20,000 and to detect rabbit IgG (Jackson ImmunoResearch Lab. Inc., Ely, UK, #111-035-003) at a dilution of 1:10,000.

The membranes were then treated with streptavidin solution conjugated with horseradish peroxidase (Sigma, Germany, #E2886) at a dilution of 1:10,000. The protein bands were revealed using 3,3′-diaminobenzidine (Amresco, Solon, OH, USA, #E733-50), and the data were analyzed in the ImageJ program. 4.5. Evaluation of the Relative mRNA Level by Quantitative PCR

Total RNA from frozen sperm was isolated using an RNeasy Micro Kit (Qiagen, Hilden, Germany, #74004) according to the manufacturer’s instructions. Reverse transcription was performed using d(T)_15_ as a primer with 50 ng of RNA. Estimation of the relative mRNA levels of the investigated genes was performed via real-time PCR with primers selected by Primer3Plus (Table 2), and the results were processed using the 2(-DeltaDeltaC (T)) method with gapdh as the reference gene [41].

### 4.5. Statistical Analysis

The results obtained were statistically analyzed in OriginPro 8.1 with ANOVA using a post hoc *t*-test with a significance level of *p* < 0.05 to assess the reliability of differences between the groups. The data are presented as the mean ± standard error of the mean (M ± SE).

## 5. Conclusions

The results obtained indicate that with brief changes in external mechanical stress, a decrease in the motor activity of mouse sperm occurs. In hypergravity conditions, after 1 h, the speed of movement decreases, as does the proportion of motile spermatozoa; in simulated microgravity conditions, after 6 h, the number of motile spermatozoa decreases. These changes in motility are associated with a change in the structure of the cytoskeleton and a decrease in the tubulin content. Changes in cellular respiration are probably an adaptive reaction to a change in the mode of motor activity.

Limitations of the study are associated with the inability to conduct research under exposure in simulated microgravity and hypergravity. Estimation of the cell respiration was carried out practically in the early period of readaptation, in 1g conditions, after simulated microgravity and hypergravity. In addition, to isolate proteins and mRNA, we froze sperm cells, which were previously briefly centrifuged. Of course, all samples, including the corresponding controls, were prepared identically, which makes it possible to study the effect of exposure, however, it would be better to carry out measurements under exposure conditions.

## Figures and Tables

**Table 1:** Primary antibodies.

Protein	Manufacturer with Catalog Number, Dilution
Cyc1 (cytochrome *c*-1, 13.5 kDa)	Abcam, UK, #ab13575, 5 μg/mL
Cox4i1 (cytochrome *c* oxidase, 16 kDa)	Abcam, UK, #ab14744, 1 μg/mL
ATP5a1 (ATPsyntase F1, 56 kDa)	Abcam, UK, #ab14748, 1 μg/mL
Gapdh (glyceraldehyde-3-phosphate dehydrogenase, 37 kDa)	Abm, Canada, #G041, 1:1000
Tuba1c (alpha-tubulin, 50 kDa)	Abcam, UK, #ab52866, 1:1000–1:50,000
Tubb4b (beta-tubulin, 50 kDa)	Abcam, UK, #ab179513, 1:1000
Cct4 (chaperonin containing Tcp1, subunit 4 (delta), 56 kDa)	Abcam, UK, #ab49151, 1.25 μg/mL
Ckap5 (cytoskeleton associated protein 5, 200 kDa)	Thermo Fisher Scientific, USA, #PA3-16835, 1:1000

**Table 2:** Primer sequences and product sizes.

Gene	Primer Sequence, Forward/Reverse (5′ 3′)	Product Size, bp
Cyc1	GTGGAACCCTGGAACCCATA/CAAACAGTGCTGCCAGGTTTT	106
Cox4i1	CTTCCCTGATTCCCGCGATG/ACACTCCCATGTGCTCGAAG	208
ATP5a1	GGCAACCACAAGGTCGATTC/CGGACGACTGGCACAAAATG	241
Gapdh	TCCCAGCTTAGGTTCATCAGG/ATGAAGGGGTCGTTGATGGC	165
Tuba1c	GGCTCGCCTAGATCACAAGT/CTCATCGTCTCCTTCAGCACT	172
Tubb4b	GAGCGTCGGTTGTAGCACTC/GATCAATGCCATGCTCGTCG	174
Cct4	TGTCTCGACCTGTGCAACTG/GTAGCTGTGGCTGGGTCAAT	151
Ckap5	GCTTGGGCAGAACAAACTGG/AGCATCTTGGGCCTTCTTCC	225
